# Book Reviews

**Published:** 1808-03

**Authors:** 


### CRITICAL ANALYSIS
OF THE
### RECENT PUBLICATIONS
ON THE
### DIFFERENT BRANCHES OF PHYSIC, SURGERY, AND MEDICAL PHILOSOPHY.

---

*Treatise on Pulmonary Consumption; in which a new View of the Principles of its Treatment is supported by original Observations on the Disease. To which is added, an Enquiry proving that the Medical Properties of the Digitalis, or Fox-glove, are diametrically opposite to what they are believed to be.* By JAMES SAUNDERS, M. D. one of the Presidents of the Royal Medical and Royal Physical Societies of Edinburgh. 8vo. pp. 318.

IN a Preface, we are told that the Author, anxious to be well informed concerning Phthisis Pulmonalis, consulted many of the writers on that subject, but was disappointed in finding that "every thing relative to that affection was involved in obscurity and confusion."

"One could scarcely perceive, says Dr. S. from their writings, that authors had ever attempted to relate the symptoms in the order of their concurrence, and consistently with the changes which succeed in the constitution; and this probably is the chief cause, that they do not agree with regard to its nature, though of bodies deprived of life by consumption an infinite number has been examined with great patience and anatomical discrimination.

"The nature of the disease not being ascertained, its causes, and the relation which it bears to other diseases, could hardly be pointed out with precision; accordingly, the causes of consumption have been confounded with consumption itself; and there is not one nosologist who has reason to be satisfied with the place assigned to it even in his own arrangement.

"Where science is imperfect, specious opinions too often usurp the authority of sound principles, and the proceedings of the artist are dictated by caprice, supposition, or fancy. This is strikingly exemplified in the treatment of phthisis pulmonalis. Here every one has some favourite remedy, which he prescribes without regard to age, sex, peculiarity of constitution, or period of the disease."

The author then, with becoming modesty, assures us he is far from presuming that he can accomplish the necessary reformation, but hopes that his own efforts may have some effect in producing a more systematic investigation, and consequent improved treatment. He then apologizes for the freedom with which he has used several illustrious names, acknowledging that in many instances the assistance he has received from them, enabled him to detect their errors; as by the light of the sun we are enabled to discern spots on its disk.

The

The second part of the work, being an experimental enquiry concerning digitalis, or fox-glove, has engrossed much of the author's time and attention. He has selected with great care the result of the facts ascertained by himself and other professional men.

"The cases detailed and opinions advanced, have, in their progress, been annually discussed in the Royal Medical and Physical Societies of this city; during which opportunities, I availed myself of every useful suggestion made by any of the ingenious members of those invaluable institutions.

"While occupied with this inquiry, facts presented themselves which seemed to me not only to improve our knowledge of the nature and treatment of consumption, and explain several paradoxical tenets in physiology and pathology, but to be importantly illustrative of the nature of hæmoptysis, hydrocephalus, anasarca of the lungs, and dropsy in general."

The work itself commences with a History of Phthisis Pulmonalis. This is in many respects well drawn, but the author has omitted to distinguish the different species of the disease. Hence the symptoms of all are accumulated in a series, as if all were to be expected in every case. In the Section containing "A detail of symptoms indicating the presence of mortification," we expected to have had some proofs of this affection of the lungs from dissection. We have indeed seen, in the advanced state of the disease, part of the lungs themselves expectorated ; but this occurs only from suppuration separating certain portions of a substance so cellular, and not from that change which the author seems to consider a sequela of high inflammation.

The Second Chapter is "concerning the nature of phthisis pulmonalis." In the first Section of this we meet with some good remarks on the various appearances of the lungs in those who dye phthisical; but, as before remarked, we have nothing to characterize the symptoms during life under these different changes. The next Section, "on the nature of the disease, and some of the discordant notions of Authors," we shall transcribe, that the Reader may judge how far the Author maintains the same modest style of expression as his Preface promised.

"When the symptoms during life warn us that local inflammation is present, and when in the dead bodies of those in whom such symptoms occurred, we discover the well known effects of inflammatory action, the inference is plain, that phthisis pulmonalis is an inflammatory affection of the lungs.

"But whatever be the state of the lungs after death, if the symptoms of inflammation were not present during life, the disease cannot have been one of an inflammatory nature.

"Whether there are any other than inflammatory consumptions of the lungs, shall be considered after we have inquired into some circumstances which principally concern that in which inflammation is certainly present.

"That phthisis pulmonalis, however, is generally an inflammatory affection of the lungs, which produces the destruction of their substance, substance, is an idea so definite and obvious, that one might have expected writers to be almost unanimous concerning the nature of the common form of this pernicious malady, which must have arrested the inquisitive attention of the wise, as well as interested the feelings of all mankind from the first dawnings of sympathy and philosophy.

"But there is scarcely, in the records of science, a better instance of how far we may be led from the very object of our research by adventitious circumstances, and the want of precise and definite terms. Though indeed every well educated person knows pretty precisely, that phthisis is a certain affection of the organs of respiration, yet, in the writings of physicians, the utmost confusion prevails with regard to all the circumstances of it: they scarcely, even now, agree that phthis pulmonalis is a disease of the lungs!

"In the judgment of Dr. Cullen, and, as far as I know, every other author of modern times, no change, ulceration, abscess, nor wasting of the substance of the lungs constitutes phthisis, unless hectic fever is present; accordingly persons, as we have seen, have died of consumed lungs without having been affected with pulmonary consumption!

"Dr. Brown, the celebrated antagonist of Dr. Cullen, asserts, that persons have died of *pulmonary consumption*, though their lungs remained *perfectly sound!*

"Dr. Darwin, if I understand him, thinks consumption of the lungs a disease of increased *sensation* without *feeling!*

"Dr. Rush, of Philadelphia, maintains, that consumption of the lungs is a *primary* disease of the *whole* system.

"But such conceits are not altogether of the present day; Willis, a celebrated physician of the seventeenth century, asserts, that *pulmonary consumption is not the ulceration or corruption of the lungs*, but a general wasting of the body from their mal-conformation.

"Argenterius, of the sixteenth century, asserts, that *great wasting* of the body is *only a tendency* to consumption, unless the lungs are ulcerated.

"And if we go back to records of ancient date, besides opinions similar to those now mentioned, we find, that consumption of lungs is a distillation from the head."

In the succeeding section, the author indulges a playfulness which we shall only hint, not doubting, that should his work come to a future edition, he will see the propriety of omitting every objectionable part. Much fewer words would have been sufficient to shew, that "we must attach precise ideas to all important words, if we wish to avoid important errors;" and also that the disease called phthisis pulmonalis, should be a disease of the lungs.—We are not, indeed, quite prepared to admit that it must be a wasting of the lungs. It is enough for us, if the lungs are diseased, and the body wasted. But whilst we give the author credit for the justice of his remarks, we cannot help wishing that he had kept them them always in view. In the succeeding chapter, on pre-disposition to, and causes of, inflammatory phthisis pulmonalis, we are told, "that the body is rendered obnoxious to phthisis by hereditary taint, the proofs are numerous." Now, to our conception, there is not a more indefinite word in physiology than a *taint.* We leave our author to study its etymology, but we are anxious for some appropriate meaning; and when we consider that the present word may be applied to a smell, to the effects of a poison, either floating in the air, or producing its effect on a part, or the whole of the living body, and that in every meaning, excepting the first, it is figurative. When we consider too that the *subject* makes the *word* highly important, we cannot help regretting that such a word should have been introduced on such an occasion.

In the progress of this chapter, the author, after objecting to all the other causes of pre-disposition to inflammation in the lungs, seems at last to satisfy himself with remarking, that the lungs, from their weakness, are unable to prevent the influx of the blood. The effects of this would by some be called congestion; but we are not disposed to enter into any controversy on this distinction.

In the following section, "on the causes of phthisis pulmonalis," the author proceeds as before, to review some of the *hypothetical causes.* On this occasion, we cannot help noticing the great disposition of the Gentlemen of the Edinburgh School to find fault with that illustrious character to whom that university owes so much.

"Dr. Cullen, (says our author,) thinks phthisis the effect of a certain indescribable acrimony! It is strange that Cullen, after having carried to extravagance the hypothetical spasms of Hoffman, and laboured to explode the humoral pathology with that of the vis medicatrix naturæ, should every where blend these doctrines with that of his favourite spasms. What can any one understand, in such circumstances, by the word acrimony? An empty sound! With other most respectable authors of every age, he enumerates among the causes, hæmoptysis, catarrh, asthma, suppuration of the lungs from pneumonia, and ulcerating tubercles. Now the first three, so far as this disease is concerned, are symptoms of it; and how is the last *cause,* viz. *ulceration of the lungs,* to be distinguished from a part of the disease."

Without following Dr. Saunders through the objections he makes to the opinions of other writers, we shall just remark, that his own opinion seems some how most unaccountably shortened. Open fire places are the only cause he assigns; and he even expresses a wish, if a board of health were appointed, which might have a power to regulate the economy of buildings, that they would enforce the warming of apartments not destined for culinary purposes, with steam, the temperature to be regulated by the thermometer. As we are now enjoying the luxury of burning our cheeks by a comfortable fire side, our author must not take it amiss amiss if we feel very jealous from the apprehension of being deprived of it.

In this short account of the first division of the work, we have endeavoured to be impartial, how much soever we may differ from Dr. Saunders; we shall offer a short abstract of the remainder, that we may find room for the important facts contained in the second part.

The fourth chapter, on the diagnosis of phthisis pulmonalis, comprehends; 1st, The chief affections, which in their symptoms resemble phthisis pulmonalis. 2ndly, An enquiry whether the disease may be known by the expectoration of pus, &c. 3rdly, Whether hectic fever is a decisive proof of the existence of phthisis.

The fifth chapter is on nosological arrangement, in which Dr. Cullen is again introduced.

The succeeding chapter contains "a view of the nature and treatment principally of phthisical affections not to be considered as inflammatory." In this, the author, after shewing that Dr. Cullen's different species are only different stages of the same disease, makes three species; the first, the inflammatory, of which we have already endeavoured to explain his opinions. "The second, independent of inflammation, and the consequence of diminished vitality. The third, in which the two former are combined, begins in a state of torpor, and becomes inflammatory."

Under the second head are enumerated many anomalous affections, taken from the dissections of Morgagni, and other faithful anatomists, as well as Dr. S's own observations. The third relates principally to scrofulous tubercles, and other similar affections."

The seventh chapter is on the treatment of the first, or inflammatory stage of phthisis pulmonalis. The eighth, on the treatment of the second, or suppurative stage. The ninth, on the treatment of the third, or last stage, which our author considers as the gangrenous, or suppuratory stage. In all these the reader will meet with practical remarks, connected with the theories, some of which we have endeavoured to compress, or have transcribed, that we may not be accused of misrepresentation or severity.

The second part of the book, containing an enquiry concerning the medical properties of a most important article in the *Materia Medica*, is highly deserving attention; and we give our author credit for the industry he discovers in his numerous experiments, as well as the unexceptionable authorities by which they are supported. These authorities are numerous, and the cases still more so. We should have complained of the tediousness of perusing them, had we not recollected, that to oppose the high opinion entertained of such names as Darwin, Beddoes, and others, it was absolutely necessary to stand on very firm ground. We shall therefore offer the result of our author's remarks, assuring our readers, that we have no reason to doubt the accuracy of those cases from which his inferences are formed.

"It

"It has been demonstrated, (says Dr. S.) that each small dose of the digitalis taken by a person in health, increases the force and frequency of the pulse; and if the doses are repeated, that they will induce an inflammatory action of the system; that also in disease, the first effects of digitalis exhibited in small doses, are to increase the force and frequency of the pulse; to excite and maintain that degree of action during which sores assume the action of healing; to promote the process by which effusions are removed from any of the cavities or parts of the body; to enliven the mind and improve the powers of voluntary motion; to invigorate digestion and increase the evacuations by the skin and by the urinary organs; in the mean time, the pulse is gradually attaining a febrile activity, so that from 70 or 90, if the use of the medicine is incautiously persevered in, the pulse shall be raised in a short time to 120, 130, or any number between these and 150.

"The stomach begins to be disordered, giddiness, beating of the vessels of the head, restlessness, general uneasiness, heat, and pains in different parts of the body, are superadded.

"Though the use of the medicine is now discontinued, these febrile symptoms may continue undiminished for four or five days; generally, however, in the space of twenty-four hours, and often much less, after their accession, the pulse sinks to 120, 110, 100, very irregular in the repetition and strength of its beats; the pulse sinks still more; the mind droops; oppression about the præcordia, and nausea, are very severe; vomiting is added, but gives no relief; at this time there are often an increased flow of saliva, diarrhœa, a very copious discharge of limpid urine, and considerable clammy moisture of skin, or even profuse sweating; the countenance is pale, and characteristic of despair. The patient may continue in this state for one, two, or even four hours, when all the distressing symptoms abate; but the pulse does not rise immediately again, but will continue to sink, and in a few days will be down to 50, 40, or even 30, and perhaps lower: this series of events I myself have witnessed; but much worse than those are recorded by Withering, whose treatise on the fox-glove is a valuable specimen of truth and candour.

"With regard to the pulse alone, during the use of the digitalis, we may observe, that its beats are gradually increased in force and frequency for some time, after which they speedily sink below the usual medium of health, and this increase and diminution follow sooner or later in proportion to the quantity of the medicine and the excitability of the system.

"The increase of force and frequency in the pulsations is greatest and most rapid during the use of digitalis in persons pre-disposed to, or affected with active local inflammation, and most speedily when either parts, previously sound, or those ulcerated, are proceeding, or are brought back to healthy suppuration, in which instances the digitalis and disease act with united violence.

"In persons, on the contrary, affected with dropsy or collections of of matter from degenerating ulcers, the pulse is invigorated, rendered steady, and, diminishing in number, returns toward the standard of health in proportion as the effused fluid or puriform matter is removed; but this diminution is very different in its nature from that which succeeds the too liberal or too long continued use of the medicine; the former is the effect of the irritating cause being removed, the latter of the powers of life being exhausted by its action; the one salutary, the other pernicious.

"When the system is in a complete state of asthenia, or when the phlogistic diathesis has been entirely subdued by the abstraction of blood, the digitalis does not, previously to the diminution of the number of the pulse, produce that great acceleration of beats with the inflammatory violence which is above pointed out: a person in such a state, and dropsical, with his pulse from 120 to 130, feeble and irregular, may be ordered to take from 10 to 15 gtt. thrice a day, with this precaution, to discontinue their use if headache or sickness supervene; at the end of eight days, the health will be improved, the swelling diminishing, and the pulse 120, 130, or even more, strong and regular; at the end of a fortnight, we shall find the dropsical swelling of the abdomen and limbs removed, all the vital and animal functions invigorated, and, though none of the symptoms of debility or exhaustion have intervened, the pulse reduced to about 72, and full; in pneumonia likewise, after the pulse has been reduced to between 80 and 90, and the phlogistic diathesis completely subdued by venæsection, great breathlessness and debility are experienced; during the use now of a few drops of the tincture of digitalis given twice or thrice a day, the vigour both of the respiration and of the general system will return, and in the course of six or ten days the number of pulsations will be increased to between 100 and 110; when neither preceded nor accompanied by any disagreeable symptoms, they may speedily diminish to between 50 and 60, strong and irregular*.

The section contains some observations not altogether new, but too little attended to on the peculiar effect produced on certain organs by particular substances.

In the following section the opinions of various authors are examined, to shew that they are often inconsistent with the facts related by themselves, and that those facts confirm our author's theory. The causes of these mistaken opinions are investigated in the next chapter. Two other sections complete the work; the first, on the comparative effects of digitalis; the last, on the administration of it. Both contain important observations, which our limits will not

* "There are many very useful observations on the powers of digitalis, in a very ingenious Treatise on the Origin, &c. of Consumption, lately published by Dr. John Reid of the Finsbury Dispensary, London. I would have frequently availed myself of this author's remarks, had I seen his treatise while I was composing this work."

not permit us to enlarge upon; but which we recommend to the perusal of our readers.

By the above remarks, our readers will perceive that we are not satisfied with the theories contained in the first part of the work: yet we wish it to be understood, that many remarks on the writings of others, however exceptionable on account of their severity, are well founded; and that the whole is interspersed with useful observations.

Of the Second Part, we cannot forbear again to express our obligations to the author.

---

### *Edinburgh Medical and Surgical Journal, for January* 1808. *No. XIII.*

[ Continued from our last, page 181. ]

Art. 5.—*Case of extensive Injury done to the Canal of the Urethra.* By Kinder Wood, Member of the Royal College of Surgeons, London.

This is an useful report; but the practical treatment suggests nothing to the experienced surgeon, excepting the manner in which it might have been improved. We mean not by this to reflect on Mr. Wood's judgment; because it is well known that few cases occur, for the first time, without leaving some impression on the mind of the practitioner, that, on a similar occasion hereafter, he might improve the treatment. In our opinion, Mr. Wood would have done better, if he had not interrupted the flabby granulations which arose at first. In subjects previously in health, mortification is often succeeded by spongy granulations; which rise very quickly, and are afterwards consolidated. In this way, we should have expected the best chance of the perfect restoration of the urethral canal.

Another objection appears too obvious to require any remark. "The first catheter," we are told, "remained in the bladder for eighteen days, when it was withdrawn by the patient; it was immediately introduced, and remained nine days longer. The second catheter remained for the space of five weeks, without any symptom coming on, indicating a necessity for its removal; when withdrawn, both catheters were broke, where they had lain under the arch of the pubis; the latter, particularly, was acted upon by the urine, and rendered very brittle; its whole surface was full of fissures, and its elasticity, in a great measure, destroyed."

Art. 6.—*A Case of Tetanus successfully treated with the Cold Affusion.* By D. Arnoldi.

Art. 7.—*A Case of the Cancer of the Penis extirpated with success.* By Mr. Thomas Machell, Surgeon, Wolsingham, Durham.

In this case Mr. Machell, by a judicious and bold operation, carefully amputated all the diseased part, though some of it was very deeply seated. In dressing the wound, it was found that the adhesive hesive plaster, spread on tin-foil, kept its figure so well, when applied round the new orifice, as to protect the cut surface from the irritation of the urine.

ART. 8.—*Biographical Sketch of* DR. PERCIVAL.

Dr. Percival's son having already prefixed a life to an edition of his father's works, we are not perfectly aware of the intention of this sketch; unless it was thought a good opportunity of introducing the following little pithy sentence.

" It appears," says the biographer, " that he [Dr. Percival] had it in contemplation, at one time, to offer himself a candidate for a fellowship in the London College of Physicians; having the natural wish of every man, whose success assures him that he has some merit, the laudable ambition of being associated with the most eminent of his contemporaries. Some feelings of personal attachment to some members of that royal corporation, might be an additional inducement for his wishing for the honour of being chronicled with them; but the honour was not obtained; and it ceased to be coveted, when the private motive was done away; so that Dr. Percival's name floats down the stream of time with those of Fothergill, Dobson, Darwin, Currie, and others, who will not be forgotten, although an English university did not contribute, in any way, to save them from oblivion."

This may be truly said to be *all fair;* and we heartily wish this mutual jealousy, as long as it exists, may extend no further, than for each party to produce its own worthies, and endeavour to increase their number.

ART. 9.—*Observations on the Operation of Lithotomy.* By ALLAN BURNS, Member of the Royal College of Surgeons, London, and Lecturer on Anatomy and Surgery, Glasgow.

This paper is fitter to be discussed in the theatre than in a journal.

A paper, by the Inquirer, " On the present State of Medical Science in Germany," closes this part of the number. It contains nothing new; but is written with more spirit than most of those, which have appeared under that title in the former numbers.

Among the medical news is the following paper, which we shall transcribe, not only on account of its importance, but because the revival of the cold affusion, in fevers, has been disputed by some, and variously attributed by others.

*Letter to* A. DUNCAN, jun. M. D.

" SIR,

" This paper, together with that on the external use of cold water in malignant fever, was written in 1769—1779, and sent to Dr. John Fothergill, for the Medical Society of London, in the beginning of the year 1779. It was read several times; at first ordered to be printed, afterwards postponed, and laid amongst their archives.

" It

"It is probable that they thought this practice so rash and daring, that they would not give their sanction to it.

"The Society was soon afterwards dissolved by the death of Dr. Fothergill, Dr. Hunter, and Dr. Solander; and I, with some difficulty, recovered my papers from Dr. Thomson, Secretary to the Society.

"Dr. Simmons published my paper on fever in 1786, but this paper on the Small-pox never saw the light.

"I sent it, with others, to Dr. Currie, at Liverpool; it is the same he alludes to in the second volume of his Medical Reports.

"I beg leave to present you with this copy, that, if you think proper, give it a place in your useful Medical Journal.

*Edinburgh, Sept.* 15, 1807.

"*On the External Use of Cold Water in the Small-pox. Communicated to Dr.* Fothergill, *and read by him to the Medical Society of London,* 1779. *By* William Wright, m. d. f. r. s.

"In the year 1768, the small-pox was in a manner epidemic in Jamaica; it proved fatal to a number of people who took it in the natural way, but only to a few who were inoculated and properly treated.

"This disease became general about the months of April, May, and June, in the parish of St. James. Such as had the disorder in the natural way, had a load of pustules, and often of the confluent kind. Sydenham's cool method of treatment was called to our remembrance, by the success of the Messrs. Suttons, and of Dr. Dimsdale. But although a liberal use of cold drinks were allowed the sick, little benefit could be expected from cool air, in such a climate and season of the year.

"It is well known, that the quantity and quality of small-pox, depends on the duration and violence of the eruptive fever; any expedient, then, to mitigate the one, would, of course, render the other more favourable.

"The maroon negroes in Jamaica, and some nations on the coast of Guinea, have a custom of plastering the bodies of such of themselves as are taken ill of the small-pox, and especially during the eruptive fever, with wet clay, and with such good effects, as determined me to try the cold bath.

"So soon as a person was seized with the variolous fever, whether from inoculation or otherwise, I caused an assistant to throw cold water on their naked bodies every four or six hours. The consequence was a truce from the fever, from the headach, and pain in the back; a glow succeeded, with a kindly perspiration. The eruption, after this, was for the most part favourable.

"In other cases, where the small-pox had made their appearance, and by their quantity, and the continuance of the fever, a confluent pock was apprehended, the cold bath not only abated the fever, but diminished the number of pustules, and the patients went through the disease easier. I do not recollect more than one pers n person, out of five hundred, treated in this manner, but what agreed perfectly well with the cold affusion.

"So soon as the eruption was completed, and the fever gone, I desisted from the external application of cold water; I kept my patients in cool air, and allowed them cold water, through the whole course of the disease.

"The secondary fever was prevented, or greatly mitigated, by timely purging the patient; and so soon as the pustules were at the height, by discharging the contents by a needle, or some sharp-pointed instrument; by the bark, and sometimes epispastics. But where the fever run high, antimonial wine, or James's powder, was given; but in common cooling laxatives, small doses of emetic tartar, with or without opiates, were sufficient; and, lastly, the bark and port wine."

*Extract of a Letter from Dr. Gairdner, dated St. James's, October 24th,* 1778.

"The small-pox made their appearance in this parish, and it became necessary to inoculate the negroes on Green-park, and Castle Weemyss estates, and also those of the small settlements. The practice was the same as baron Dimsdale recommends; only, during the eruptive fever, we used the cold bath; that is, we dashed cold water on their heads and in their faces, which had a remarkable effect in giving immediate ease; it carried off the fever, and no doubt lessened the eruption.

"At Castle Weemyss estate, when the negroes were feverish, I made them go in below the spout of water. They were so much pleased with its good effects, that they often went below it of their own accord. All of them had the small-pox remarkably easy. One hundred and twenty of them were inoculated without any preparation; they were kept at easy work during the whole time of the disease, and were all able to go to their usual employment in 14 days after being inoculated.

"The same success did not attend the negroes on the small settlements, which I think was owing chiefly to their not using the cold bath."

